# Biopolymers Used for Receptor Immobilization for Nickel-Detection Biosensors in Food

**DOI:** 10.3390/mi14081529

**Published:** 2023-07-30

**Authors:** Liliana Anchidin-Norocel, Wesley K. Savage, Roxana Gheorghita, Sonia Amariei

**Affiliations:** 1 Faculty of Medicine and Biological Sciences, Stefan cel Mare University of Suceava, 720229 Suceava, Romania; roxana.puscaselu@usm.ro; 2 Integrated Center for Research, Development and Innovation in Advanced Materials, Nanotechnologies, and Distributed Systems for Fabrication and Control, Stefan cel Mare University of Suceava, 720229 Suceava, Romania; 3 Faculty of Food Engineering, Stefan cel Mare University of Suceava, 720229 Suceava, Romania

**Keywords:** biopolymers, enzyme, screen-printed electrode, nickel ions, optimization, urease

## Abstract

Food is humans’ main source of nickel intake, which is responsible for the prevalence of allergic contact dermatitis and other pathological afflictions. While robust, the classical methods for nickel detection—atomic absorption spectrometry and inductively coupled plasma mass spectrometry—are expensive and laborious; in contrast, modern methods that utilize sensors—of which most are electrochemical—have rapid run times, are cost-effective, and are easily assembled. Here, we describe the use of four biopolymers (alginate, agar, chitosan, and carrageenan) for receptor immobilization on biosensors to detect nickel ions and use an optimization approach with three biopolymer concentrations to assay analytical performance profiles. We measured the total performance of screen-printed carbon electrodes immobilized with the biopolymer–sensor combinations using cyclic voltammetry (CV). Voltammetric behavior favored the carrageenan biosensor, based on performance characteristics measured using CV, with sensitivities of 2.68 (for 1% biopolymer concentration) and 2.08 (for 0.5% biopolymer concentration). Our results indicated that among the four biopolymer combinations, carrageenan with urease affixed to screen-printed electrodes was effective at coupling for nickel detection.

## 1. Introduction

The immobilization of biopolymers on the surface of electrosensors has demonstrated they are promising candidates for a new generation of sensors. Electrochemical sensors have become important tools in medical diagnostics and health care, environmental monitoring, food safety, and now for COVID-19 detection [1,2,3]. This is due to their high specificity, sensitivity, and capacity for real-time analysis coupled with their speed and low cost of production. The general workflow for electrochemical biosensors is that a protein macromolecule (e.g., an enzyme) is reacted with a target element, inducing a chemical change that causes a signal measured using a transducer [4,5]. Enzymes are commonly used in biosensors because of their innate biological relevance for binding to a variety of substrates relevant to public health. More recently, sensor technology has seen improvements with the immobilization of enzymes on supporting materials that enhance biosensor response and utility [6,7,8].

Entrapment is a method widely used for the sensor immobilization of enzymes, antibodies, and nucleic acids. It involves the preparation of an electrolyte solution containing both a monomer and a biomolecule, followed by the electro-polymerization of the components in the solution. This method produces a polymer film containing biomolecules formed at an electrode surface. This technique is useful because it leads to strong adhesion between a biomolecule and a biopolymer film in a single step, and generally leads to biosensors with greater sensitivity and functional duration [9].

Biosensors can be constructed with biopolymers, which are seeing wider uses in the detection of specific compounds in foods and in pharmaceutical, cosmetic, and other biochemical applications. Biopolymers are economical, tunable, biodegradable, readily available, biocompatible, and biologically relevant to health applications [10]. In sensor applications, biopolymers are entrapped on surfaces by being mixed with an enzyme and crosslinked with multivalent cations in an ion–exchange reaction. This forms a lattice structure that traps the enzymes and creates ionotropic gelatins [11,12,13]. There are several biopolymers that can be used for the manufacture of biosensors, among them we mention agar, alginate, chitosan, carrageenan, and cellulose—which is an abundant natural macromolecule [14,15] —and also pectin, gelatin, and acacia gum [16].

In the past decade, chitosan, a chitin-derived polysaccharide, has been used successfully as a biocompatible matrix to immobilize biological sensing elements for biosensor construction. Chitosan is an effective enzyme immobilization matrix because of its adhesion properties, nontoxicity, and biocompatibility with many substances of analytical interest [6]. The advantages of chitosan are that it has a potent metal-binding affinity, is metabolized by some human enzymes (e.g., lysozyme), and is biodegradable [17,18,19]. Chitosan is very sensitive to different experimental parameters that affect its stability, such as the degree of deacetylation, polymer concentration, type, and concentration of acid, requiring careful procedural techniques to ensure stable reaction conditions [20].

In addition to chitosan, alginate is a useful biopolymer for various biochemical applications. It is a natural anionic polymer found in brown algae, composed of 1,4-linked α-l-guluronate and β-d-mannuronate residues arranged in linear copolymer blocks. Alginate hydrogels are formed by crosslinking the linear polysaccharide chains, which confers a longer duration on electrodes. Typical methods for alginate crosslinking to electrodes are based on three physical and chemical approaches: (i) covalent crosslinking by the addition of a reagent that promotes covalent bonds between alginate chains; (ii) thermal gelation based on temperature changes; and (iii) ionic crosslinking by the addition of cell adhesion ligands [21].

Carrageenan is a high-molecular-weight, hydrophilic polysaccharide naturally extracted from red algae. Due in part to its helical conformation, it can form a biogel from the aggregation of biomolecules. This forms a biocompatible matrix that stabilizes the biomolecules of aggregated carrageenan chains, and its porous structure allows efficient diffusion of substrates that promote enzyme rate [22]. Carrageenan has a number of advantages for use in biosensing, such as being biocompatible, non-toxic, highly viscous with high gelling capacity, and stability in a wide pH range [23].

Agar is a polysaccharide that contains agarose, a sugar with a strong gelling ability. It is acid-stable, which is important for binding samples suspended in acidic solutions, and shows no protein reactivity. It is also lower in cost compared with other materials commonly used for immobilization [24,25,26]. Agarose has been widely used in biologics, especially in nucleic acid recovery, because as a matrix it is highly porous, mechanically resistant, chemically and physically inert, and hydrophilic. Covalent crosslinking could improve these features, rendering them particularly suitable for enzyme immobilization with a wide range of methods that take advantage of chemical modification. Agar (and agarose) dissolves readily in 100 °C water, creating a useful matrix for enzyme immobilization. It is noteworthy that, contrary to other hydrophilic matrices, gels of agar and agarose do not appreciably shrink or swell with the introduction of other solutions; the 3-D architecture of the polymer net remains intact even when water molecules are driven out and substituted by other solvents [27]. Biopolymer-based sensors are inexpensive, biocompatible, sensitive, selective, and require minimal production effort. These properties open many new opportunities in various fields of research—including in public health and food safety [17]—in the detection of nickel, which is a ubiquitous element found in water, food, and many materials used in product manufacturing for a wide range of general-use applications. It is one of many heavy metals that cause allergic responses in people around the world. Across Europe, nickel allergies affect 8% to 19% of adults and 8% to 10% of younger individuals, with indications that women are more heavily affected than men. Contact with nickel can cause systematic nickel allergy syndrome (SNAS), which presents numerous symptoms including dermatologic and generalized cutaneous conditions, and systemic (e.g., fibromyalgia, headache), respiratory, and gastrointestinal symptoms, among others [28]. Dermal exposure to nickel occurs by contact with a variety of metallic-based items, common household products, and cosmetics, whereas intrinsic exposure occurs through food and water consumption, and medical and dental implants [29]. To date, much of the focus on SNAS has been on dermal contact, with less attention to nickel in food, despite this pathway also producing skin disorders and systemic symptoms. It is therefore important for public well-being to develop methods to measure nickel ion concentrations in food raw materials and food [30].

Urease is a nickel-dependent enzyme [31], and the reaction mechanism between it and nickel has been presented in different ways by researchers. There is an initial coordination of urea to the active site, which is accomplished by urea oxygen attacking the vacant coordination site on Ni^1+^. The initial mechanism involves an attack on the urea carbon by a hydroxide that is terminally bound to Ni^2+^. This leads to an intermediate that binds the two metals and can release ammonia to form secondary products [32].

Effective immobilization methods of enzymes require efficient process levels with retention of catalytic activity. The most common methods used for enzyme immobilization include polymer entrapment, affinity, physical adsorption, covalent binding, and cross-linking [33,34,35]. These methods have different characteristics that determine their usefulness for a wide range of applications and are in continuous testing and development.

In this study, we describe a methodological approach to detect nickel levels in foods with four biopolymers (alginate, agar, chitosan, and carrageenan) immobilized on urease biosensors, and use an optimization approach with three biopolymer concentrations. We measured the analytical performance profiles of the four biopolymers used for the immobilization of urease on electrodes using cyclic voltammetry.

## 2. Materials and Methods

### 2.1. Reagents

We used commercial analytical reagents to verify the efficiency of four biopolymers for receptor immobilization on electrodes: nickel sulfate (0.5–10 mg/L) to standardize measurements; an ammonium buffer solution with pH = 8 (electrolyte solution); urea stock solution (1 M) and urease (0.1%) as a receptor; the four biopolymers—agar, alginate, carrageenan, and chitosan (acetic acid solution as a solvent (0.1 M)). These were all acquired from Sigma Aldrich Chemical Corp. (St. Louis, MO, USA). Using the above reagents, we tested all four biopolymers in three concentrations: 0.5, 1, and 1.5%. We immobilized urease with each biopolymer on carbon-based screen-printed electrodes (SPEs) (DRP110, dimensions: 3.4 × 1.0 × 0.05 cm, Metrohm DropSens, Metrohm Corp., Herisau, Switzerland) for single use. The electrochemical cell consists of a working electrode—carbon (4 mm diameter); an auxiliary electrode—carbon; and a reference electrode—silver. All solutions were prepared with deionized water with a resistivity of 18.2 M (Millipore, Direct-Q 3 UV, Millipore Corp., Burlington, MA, USA). These four biopolymers were chosen in the experiments because all of them are capable to immobilized the enzyme, as was mentioned in the introduction.

### 2.2. Voltammetric Measurements

Estimation of the amount of bound urease is important for ascertaining the efficiency of immobilization methods, as it is well known that nickel ions will not interact with a receptor in low quantity/concentration.

Urease contains electroactive amino acid residues and requires two nickel ions for its catalytic activity, so it is possible that urease-SPEs may not react to the substrate and contribute to differences in anodic peaks. To correct this, we conducted repeated measures across multiple trials.

We measured voltammograms with a Metrohm Autolab bipotentiostat µStat 300 (Metrohm Corp., Herisau, Switzerland) and DropView 8400 software (v. 3.6 20B0514) for all experimental measurements. Voltammetric measurements were carried out at room temperature (22 ± 2 °C), with 50 µL of sample solutions containing the supporting electrolyte and the analyte (i.e., nickel solution). We used four types of biopolymers (agar, alginate, carrageenan, and chitosan) to immobilize the urease receptor.

### 2.3. Procedures

The four biopolymers and three concentration-based biosensors were measured using cyclic voltammetry (CV) in the potential range of −0.1 to 0.9 V, with a step potential of 0.002 V, and a scanning rate of 0.05 V/s. We tested and optimized concentrations of the four receptor combinations for functional biosensing interactions with nickel. Control experiments were carried out with immobilization of urease with biopolymers in the same condition of measurement but in the absence of nickel. We used the CV technique herein because it was effective in previous work [36]. Here we use this technique to evaluate sensor performance with different types and concentrations of immobilizing agents on carbon electrodes, which are less expensive than silver electrodes.

### 2.4. Immobilization of Receptor on Screen Printed Electrodes (SPEs)

Agar, alginate, and carrageenan were prepared in deionized water; chitosan was prepared in an acetic acid solution (0.1 M). A small drop (1 µL) of urease was pipetted on screen-printed electrode surfaces and immobilized with biopolymers (1 µL) at room temperature conditions for three hours, after which each biopolymer was measured using voltammetry (CV).

### 2.5. Characterization of Biosensor Performance

Biosensor performance was measured by sensitivity, limit of detection, reproducibility, stability, and selectivity.

#### 2.5.1. Sensitivity

Sensitivity was calculated using the basic formula below (Equation (1)):

Sensitivity = m/A
(1)

where m—slope of calibration curve (μA mM^−1^); A—area of active surface (cm^2^) [37,38].

#### 2.5.2. Limit of Detection (LoD)

The limit of detection (LoD) was defined as the lowest measured analyte concentration at which the signal was greater than three times the standard deviation of the negative control, expressed in the units used in the relevant reference [39].

Our methodological approach took these into account with stabilization procedures to normalize the results across trials.

#### 2.5.3. Selectivity

Biosensor selectivity was measured with a nickel-generated current at 10 mg/L of the sample because this concentration can be found as the maximum value in food. Evaluation of selectivity was carried out for each biopolymer used for biosensors with different concentrations using CV. The mineral elements analyzed by the same techniques and the same conditions were as follows: zinc, cadmium, iron, copper, magnesium, sodium, and calcium.

## 3. Results

### 3.1. Voltammetric Behavior of Receptors with Four Biopolymers

**Figure 1:**
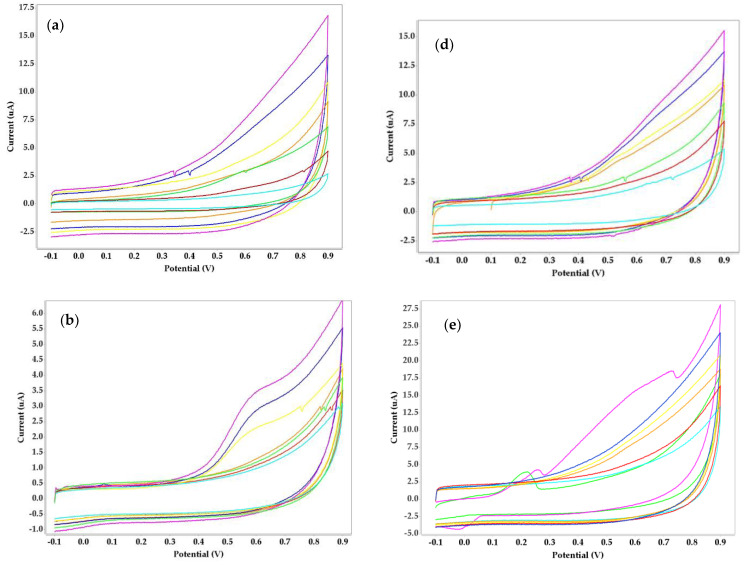

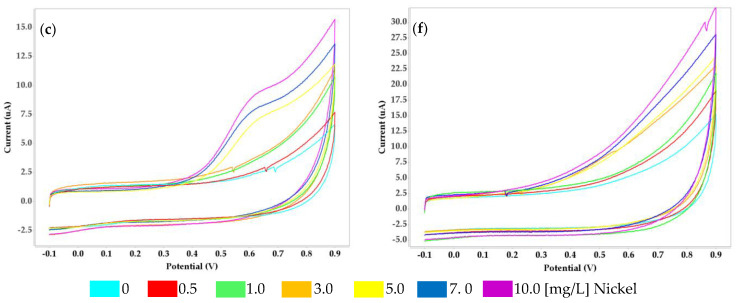
Cyclic voltammograms obtained for three concentrations of agar (**a**) 0.5%, (**b**) 1%, (**c**) 1.5%, and three concentrations of alginate (**d**) 0.5%, (**e**) 1%, (**f**) 1.5%.

The activity of urease depends on the presence of nickel. Urease interacts with nickel ions at the surface of the working electrode (urease being immobilized in the biopolymer), and the signal increases (anodic peak current) with interaction time proportional to complete coverage of the surface. Stratification or other nonspecific interactions of target analytes may then occur, leading to a reduction in the measured signal. The voltammetric behavior of four urease-immobilized biosensors showed irreversible anodic peaks at 0.65 V for 1% and 1.5% agar concentrations but no peaks were evident for 0.5%, indicating agar is less efficient at this concentration as a biopolymer for nickel detection. Cyclic voltammograms recorded for agar and alginate immobilized with urease and a nickel standard solution are shown in Figure 1. The voltammetric method intuit that higher concentrations of biopolymers are more detectable than lower ones.

**Figure 2:**
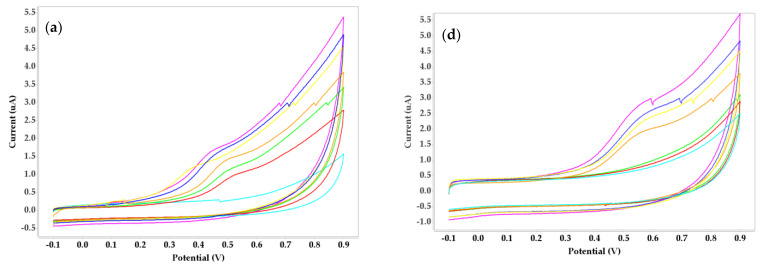

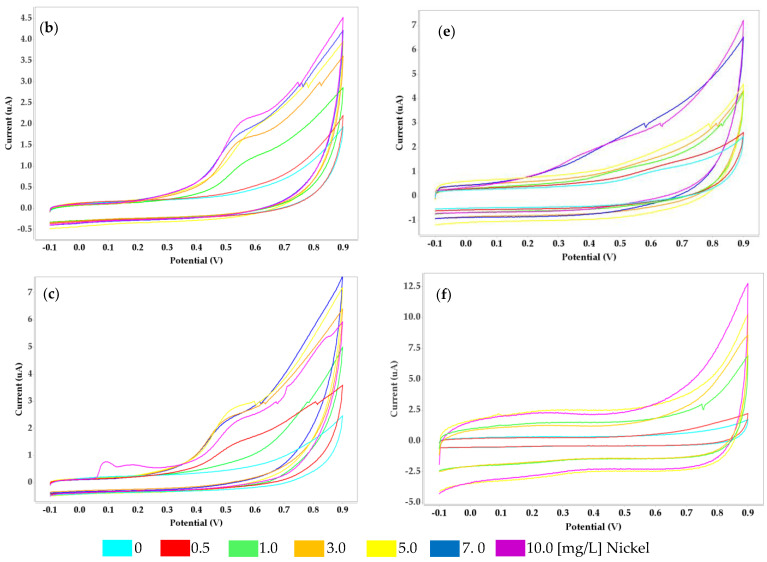
Cyclic voltammograms obtained for three concentrations of carrageenan (**a**) 0.5%, (**b**) 1%, (**c**) 1.5%, and three concentrations of chitosan (**d**) 0.5%, (**e**) 1%, (**f**) 1.5%.

No significant peaks were recovered for cyclic voltammetry at the 0.5 and 1.5% concentrations of alginate, while the 1% alginate concentration produced redox peaks for two concentrations of nickel (1 and 10 mg/L). For the carrageenan biosensor combination, all concentrations displayed an irreversible anodic peak with CV: a 0.5% concentration with a potential at 0.45 V, while the other two concentrations (1 and 1.5%) had a potential of 0.55 V (Figure 2a–c). The three concentrations of chitosan showed different voltammetric behaviors (Figure 2d–f), where visible peaks are apparent only for the 0.5% concentration at 0.6 V.

### 3.2. Characteristics of the Biosensor’s Performance

The performance characteristics (based on R^2^ of calibration curves, sensitivity, limit of detection, and selectivity) for nickel detection of the four biopolymer-based biosensors (agar, alginate, carrageenan, and chitosan, immobilized on urease-carbon SPEs) are presented in Table 1 and the Supplementary Figures S1–S4. The results for the linear regression, sensitivity, and limit of detection were obtained for the three concentrations of each biopolymer.

Based on the characterization in Table 1, the highest sensitivities (µA Mm^−1^ cm^−2^) are found in the following order: carrageenan, chitosan, agar, and alginate. The limit of detection (in mg/L) is negatively correlated (−0.805) with sensitivity, which means that the same biopolymers, techniques, and biopolymer concentrations are effective in nickel detection.

**Figure 3:**
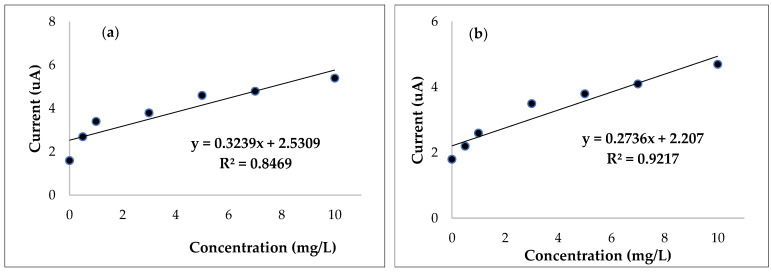
Calibration curves for carrageenan measured using cyclic voltammetry: (**a**) 0.5%, (**b**) 1%.

The most efficient biosensors, determined by the highest sensitivity and the lowest limit of detection, proved to be with 0.5 and 1% concentrations of carrageenan with urease, and the calibration curve is presented in Figure 3. The calibration curves for the other biopolymer-based biosensors are illustrated in Figures S1–S4. Even if the linear regressions of the illustrated calibration curves are in medium values, the other performance characteristics are better, which makes the R^2^ parameter not significant for optimization, although we included it.

**Figure 4:**
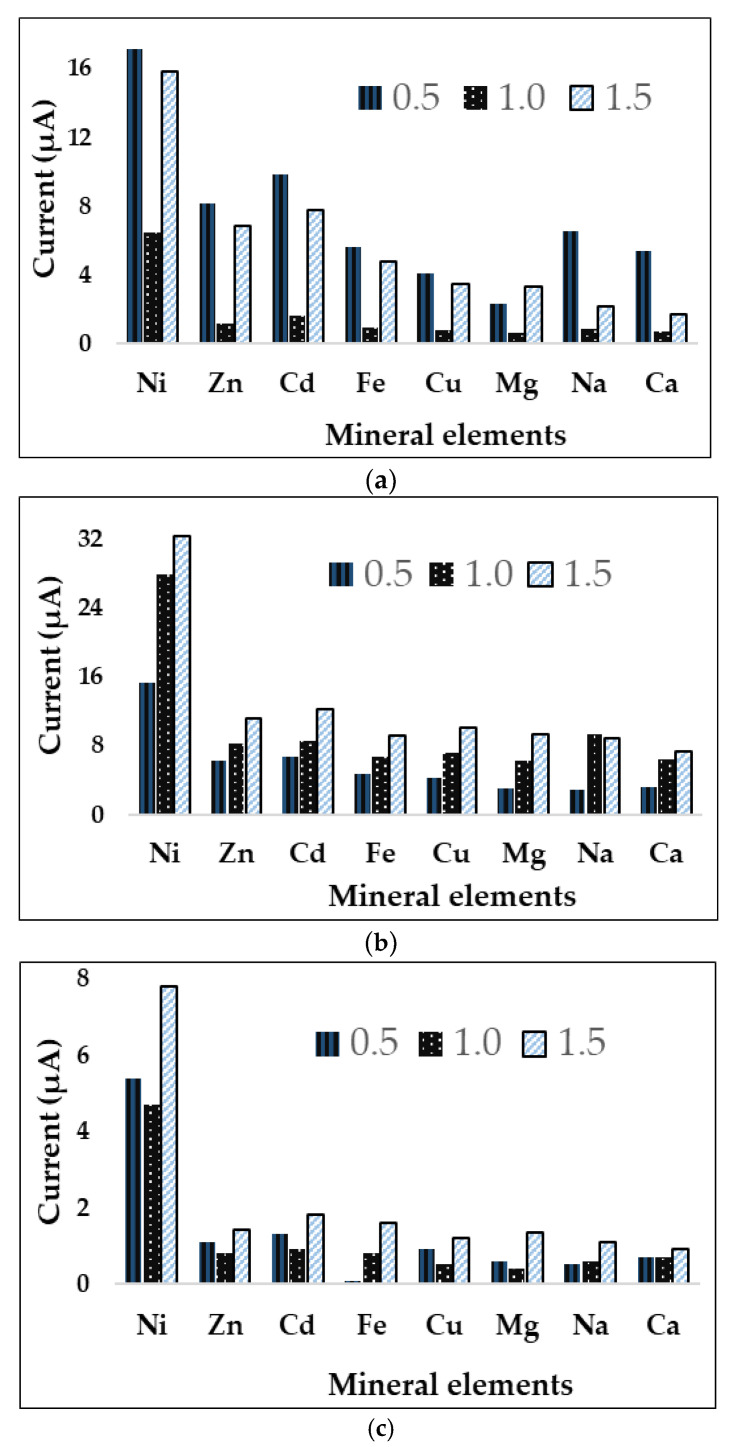

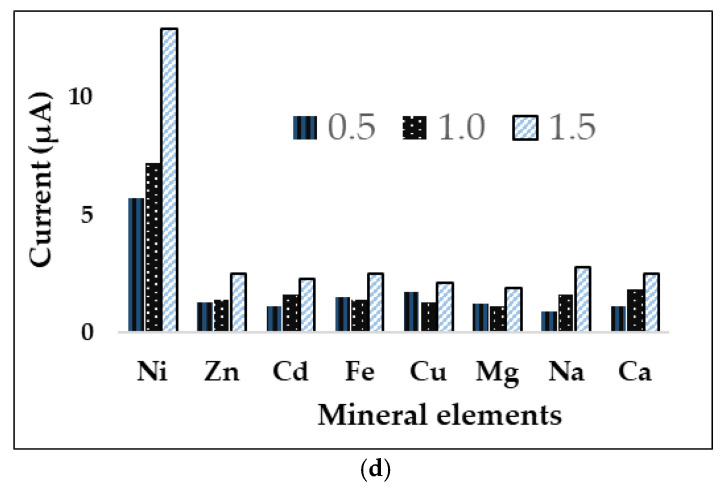
Selectivity evaluation of four biopolymer-based biosensors against the interference of mineral elements with cyclic voltammetry for (**a**) agar, (**b**) alginate, (**c**) carrageenan, and (**d**) chitosan.

We investigated the effect of competitive metal ions on the electrochemical determination of nickel content using the biosensor based on the immobilization of urease with four biopolymers. The experiments were carried out by recording changes in voltammetric behavior, both before and after adding the different metal ions, Zn^2+^, Cd^2+^, Fe^3+^, Cu^2+^, Mg^2+^, Na^+^, and Ca^2+^, into an ammonium buffer solution. The concentration of Ni^2+^ was 10 mg/L as were the other ions (Figure 4a–d). In general, the selectivity evaluation showed good values for the majority of biopolymers but was the highest for carrageenan, and demonstrates that it performed better analytically versus the other biopolymer candidates we tested.

The repeatability (n = 6) of biosensors was evaluated using the same conditions for six days. The relative standard deviation (RSD) values obtained for intra-day repeatability were 2.38% for carrageenan, 2.54% for alginate, 3.87 for agar, and 4.21% for the chitosan biosensor; inter-day repeatability was 3.62% for carrageenan, 3.84% for alginate, 4.52% for agar, and 5.46 for the chitosan biosensor. Furthermore, the reproducibility of the method (the biosensors are single-use) was determined by assessing standard deviations: 3.87% for carrageenan, 4.35% for alginate, 4.84% for agar, and 5.46 for the chitosan biosensors.

Thermal stability is an important factor determining the utility of biopolymer-based sensors, as instability or aging can vary considerably across a range of temperatures. Here, the long-term stability of biosensors was evaluated by monitoring the response in the presence of nickel in ammonium buffer solution with pH = 8 for 60 days at room temperature (∼24–26 °C), to control across different sensor combinations.

The cathodic current response decreased by a maximum of 15% after this time for all types of biosensors. This stability is due to the immobilization procedure, which provides good entrapment of the urease enzyme.

### 3.3. Optimization of Four Biopolymers and Concentrations

**Figure 5:**
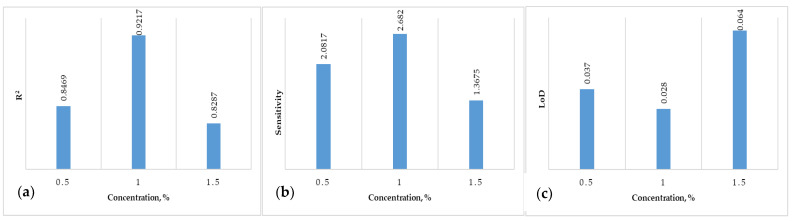
Performance parameters of three concentrations of carrageenan (0.5, 1, 1.5%): (**a**) R^2^; (**b**) sensitivity (µA Mm^−1^ cm^−2^); and (**c**) limit of detection (LoD; mg/L).

We optimized three different concentrations (0.5, 1, 1.5%) for four biopolymers (agar, alginate, carrageenan, and chitosan), analyzed for efficiency using CV. We include the R^2^ values of the results, as well as sensitivity and limit of detection (LoD) values (Figure 5). The regression analysis (Figure 5a) suggests a good fit by a two-factor (technique and concentration) interaction equation (F-value = 80.18; *p <* 0.0001). ANOVA results for the 2FI model with sensitivity and LoD were also significant (Figure 5b,c), the first with F = 145.75 (*p* ≤ 0.0001), and the second with F = 7.76 (*p* = 0.0181). Carrageenan biosensor sensitivity showed higher values for 1% concentration (Figure 5b), and the LoD values are greater for the 0.5% concentration (Figure 5c).

The optimization process displayed a desirability score of 0.883 (range from suboptimal to optimal, 0 to 1.0), indicating that the 1% carrageenan solution was the most efficient biosensor combination (sensitivity—2.6820 µA Mm^−1^ cm^−2^; LoD—0.028 mg/L) for nickel detection.

## 4. Discussion

Carbon-based electrodes are widely used in electroanalytical investigations for the development of sensors to determine organic and inorganic analytes because of their low cost, good electron transfer kinetics, and biocompatibility [36,40]; moreover, modification of the carbonaceous surface is necessary to improve the electrocatalytic potential for the oxidation of organic and inorganic hydroperoxides. Urease is a nickel-dependent enzyme found in many organisms, including algae, fungi, prokaryotes, and plants. It is very substrate-specific and catalyzes the hydrolysis of urea into carbon dioxide and ammonia. Due to these properties, urease is a biologically relevant compound in natural organisms [41]; therefore, we used it as the ligand to bind nickel with voltammetric measurements. During the reaction, urea undergoes hydrolysis, which leads to the formation of carbon dioxide and ammonia. In this process, the nickel atom is reduced, that is, it receives electrons from urea. The reduced nickel atom allows the stabilization of some reactive intermediates during the reaction, which facilitates the decomposition of urea. Electron transfer occurs between the nickel atom and urea during the reaction catalyzed by urease. Before the reaction, urea binds to the active site of urease, forming a bond with the nickel atom; thus, the transfer of electrons between urease and nickel is essential for the proper functioning of urease.

When current flows through the electrolytic cell, a potential difference is created that drives electrons to move. At the counter electrode where reduction occurs, electrons are transferred to nickel ions, causing their reduction to metallic nickel. This electron transfer allows the nickel ions to accept electrons and convert them into neutral nickel atoms, which are then deposited on the working electrode. Here, where oxidation occurs, nickel atoms lose electrons and are oxidized to nickel ions. The current facilitates electron transfer, thereby reducing nickel ions at the cathode and correspondingly oxidizing nickel atoms at the anode; thus, electron transfer in this context refers to the movement of electrons from the anode to the cathode through an external circuit, thereby ensuring the necessary redox reactions at the respective electrodes.

A previous study on nickel biosensor development reported that urease affixed to SPEs was effective for nickel detection in food samples [36]. Using the same dynamic range from the previous study, we furthered the work by optimizing receptor immobilization agents with four biopolymers types and concentrations. We observed performance improvements of different concentrations of immobilization agents, from which the best-performing biosensors are self-indicating.

The voltammetric behavior for presented biosensors indicates that the most favorable biosensor combination includes carrageenan, identified by irreversible peaks with CV. Based on performance characteristics, the highest values were obtained for carrageenan at 1% concentrations, with sensitivities of 2.6820 µA Mm^−1^ cm^−2^. This was followed by the 0.5% chitosan (2.5389 µA Mm^−1^ cm^−2^) and 1% agar (2.3800 µA Mm^−1^ cm^−2^) combinations with the urease biosensor. LoD values were highest for the same biopolymers and concentrations (0.028 mg/L at 1% for carrageenan, and 0.020 mg/L of 1% agar). These results suggest they are good candidate biopolymers in biosensors that are designed for detecting nickel in sample analytes. The advantages of this developed biosensor compared to others include: improved sensitivity for carbon SPE—via changing the biopolymers used (alginate with carrageenan)—compared to the previous study (from 0.1725 to 2.6820) [36]; and the food-specific linear range that is not present in many studies but has importance for rapid testing, especially in the case of an allergic person. These improvements may be due to the carrageenan that facilitated the development of a compact matrix and immobilized the biomolecules; thus, the porous structure allows efficient diffusion of substrates that promote the urease rate. Aspects related to carrageenan’s properties that make it suitable for entrapment on surfaces of electrodes will be investigated in future studies. An important and valuable characteristic of biosensors is their specificity. This is considered the most essential quality of any analytical sensor, as it describes its ability to differentiate between target and non-targeted biological entities in a sample. The results of these tested outcomes are encouraging because the carrageenan-based biosensor was confirmed by the optimization process as the most promising biopolymer tested. While the stability of the biosensors we examined is in continued development, Singh et al. (2008) observed that the stability of immobilized urease as a receptor reached 60 days with 20% loss, with urea decomposition being slower in the case of immobilized urease over free urease. In the same study, the optimum pH of the immobilized urease was observed to be 0.5 units more than the free urease [42]. These results point to the durability of the urease-bound biosensor for multiple applications.

The literature is lean on this topic, despite the importance of biopolymers and their advantages for the development of sensors [43,44,45,46]. Kuralay et al. (2011) reported the development of a single-walled carbon nanotube–chitosan-modified disposable pencil graphite electrode for the electrochemical monitoring of vitamin B12. The device aimed to achieve a signal enhancement of the analyte in comparison to a chitosan-modified disposable pencil with a graphite electrode. The selected molecule (vitamin B12) is a corrin-based cobalt complex, important in human physiology because its deficiency causes pernicious anemia and neuropathy [46]. Immobilization of urease was assessed in several studies that used chitosan [47], alginate [36,48], kappa-carrageenan supports [49], and agar [50]. In one study, Jack-bean-derived urease was immobilized with chitosan–alginate polyelectrolyte complexes and j-carrageenan, where the optimum pH values for immobilized urease were 7.5 and 8.0, respectively. The optimal temperatures for these free and immobilized enzymes were 55, 60, and 55 °C [49], suggesting that they function in achievable temperature ranges for regular study.

Krajewska and Piwowarska (2005) used chitosan gel membranes as a support for the covalent immobilization of Jack-bean-derived urease. The effects of the local microenvironment created by both the electrostatic potential of the polycationic support and the enzyme reaction on the inhibition of urease by phosphate buffer were investigated as a function of pH and compared with other urease competitive inhibitions. They found the kinetic behavior of chitosan-immobilized urease in the inhibition results from structural, diffusion limitation-related, and microenvironmental factors [51].

Mulagalapalli et al. (2007) reported results with immobilized pigeon pea urease, a common gelling substance, regarding immobilization with agar. They used tablet strips to mold agar tablets of uniform shape and size [50].

Biopolymer matrices have significant advantages over other materials; they are easy to prepare, biologically relevant, are of low cost, have a high immobilization capacity, and possess good mechanical stability for various biotechnological, biomedical, and agriculture applications. For nickel biosensors, there are no similar studies regarding the use of biopolymers in the immobilization process to compare our results with, leading us to continue examining the workflow described here. The performance of linear correlation coefficients will be the focus of our future work on combinations of elements in biosensor optimization.

## 5. Conclusions

Biopolymers are biocompatible and serve as an ideal matrix for the immobilization of biomolecules, and hence are widely used in the development of biosensors for various applications. Most biopolymers possess the ability to swell in aqueous solutions, helping to eliminate the diffusion barrier for analytes. Biopolymers are also amiable for the preparation of composites with various functional materials, such as carbon-based nanomaterials and metal nanoparticles, leading to the enhancement of their properties.

In voltammetric tests of biopolymer-based sensors, a current is produced by sweeping the potential applied between a reference electrode and a biopolymer-modified electrode over a range associated with the redox reaction of an analyte. This redox reaction changes the peak current, which can be correlated with analyte concentrations that provide specific quantitative analytical information. Cyclic voltammetry has the advantage of providing both qualitative information deduced from the potential location of current peaks, and quantitative information deduced from peak-current intensity. From all combinations of the four biopolymers concentrations, the most effective and useable was the carrageenan-based biosensor (at concentrations of 0.5% and 1%) based on the performance characteristics described by the irreversible peaks in with CV and the optimization results. In summary, we found that the carrageenan biopolymer bound to urease on screen-printed electrodes holds potential for the adaptive measurement of nickel in samples. Continued development with the approach we employed may yield more sensitivity in receptor development for nickel-detecting biosensors.

## Figures and Tables

**Table 1:** Analytical performance characteristics of four biopolymer-based biosensors with three concentrations, measured by efficiency (R^2^, Sensitivity, and LoD) with cyclic voltammetry.

Biopolymer	Concentration [%]	R^2^	Sensitivity [µA Mm^−1^ cm^−2^]	Limit of Detection (LoD) [mg/L]	Stability (Weeks)
Agar	0.5	0.9495	0.5375	0.068	3
	1	0.9512	2.38	0.02	
	1.5	0.9002	0.861	0.026	
Alginate	0.5	0.9222	0.8102	0.094	5
	1	0.9809	0.5719	0.098	
	1.5	0.9376	0.4951	0.099	
Carrageenan	0.5	0.8469	2.0817	0.037	6
	1	0.9217	2.682	0.028	
	1.5	0.8287	1.3675	0.064	
Chitosan	0.5	0.9778	2.5389	0.052	3
	1	0.9096	1.5611	0.074	
	1.5	0.8411	0.6836	0.086	

## Data Availability

The data presented in this study are available upon request from the corresponding author.

## Supplementary Materials

The following supporting information can be downloaded at: https://www.mdpi.com/article/10.3390/mi14081529/s1, Figure S1: Calibration curves for the agar biopolymer chemosensor measured by cyclic voltammetry: (a) 0.5%, (b) 1%, (c) 1.5%; Figure S2: Calibration curves for the alginate biopolymer chemosensor measured by cyclic voltammetry: (a) 0.5%, (b) 1%, (c) 1.5%; Figure S3: Calibration curves for the carrageenan biopolymer chemosensor measured by cyclic voltammetry: (a) 0.5%, (b) 1%, (c) 1.5%; Figure S4: Calibration curves for the chitosan biopolymer chemosensor measured by cyclic voltammetry: (a) 0.5%, (b) 1%, (c) 1.5%.
