# Supplementary material for: Biopolymers Used for Receptor Immobilization for Nickel-Detection Biosensors in Food
Source: Micromachines (Basel). 2023 Jul 30;14(8):1529. doi: 10.3390/mi14081529 (PMC10456834; doi:10.3390/mi14081529)
Supplement: Supplementary file 1 [file micromachines-14-01529-s001.zip › micromachines-2479700-supplementary.pdf]

micromachines

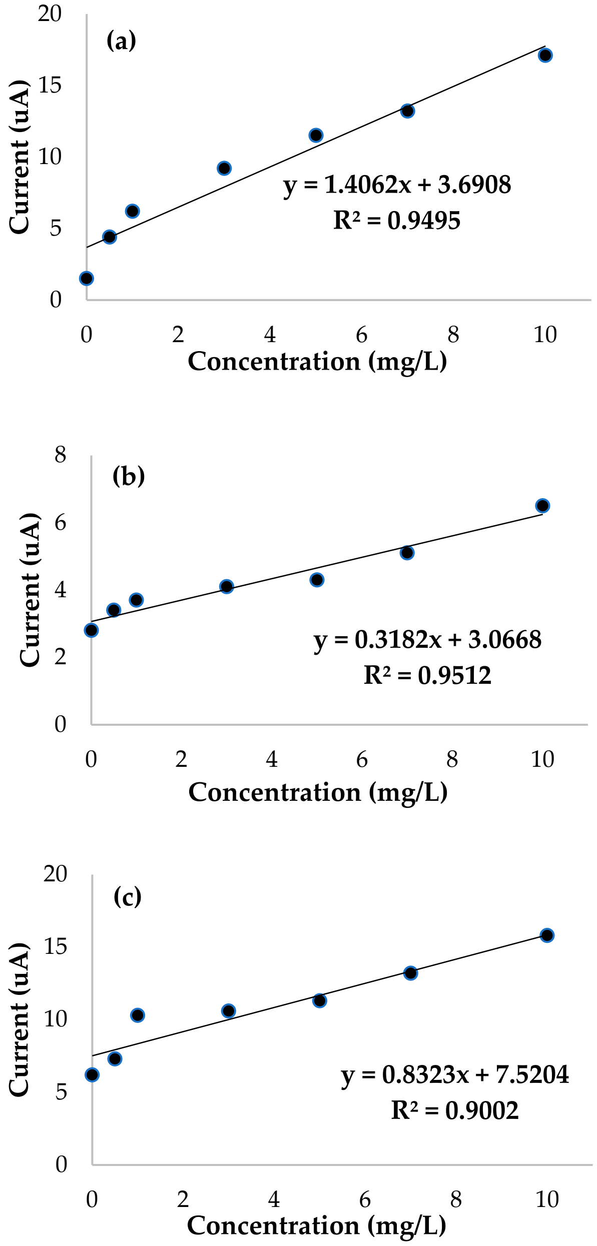

Figure S1. Calibration curves for the agar biopolymer chemosensor measured by cyclic voltammetry: (**a**) 0.5%, (**b**) 1%, (**c**) 1.5%;

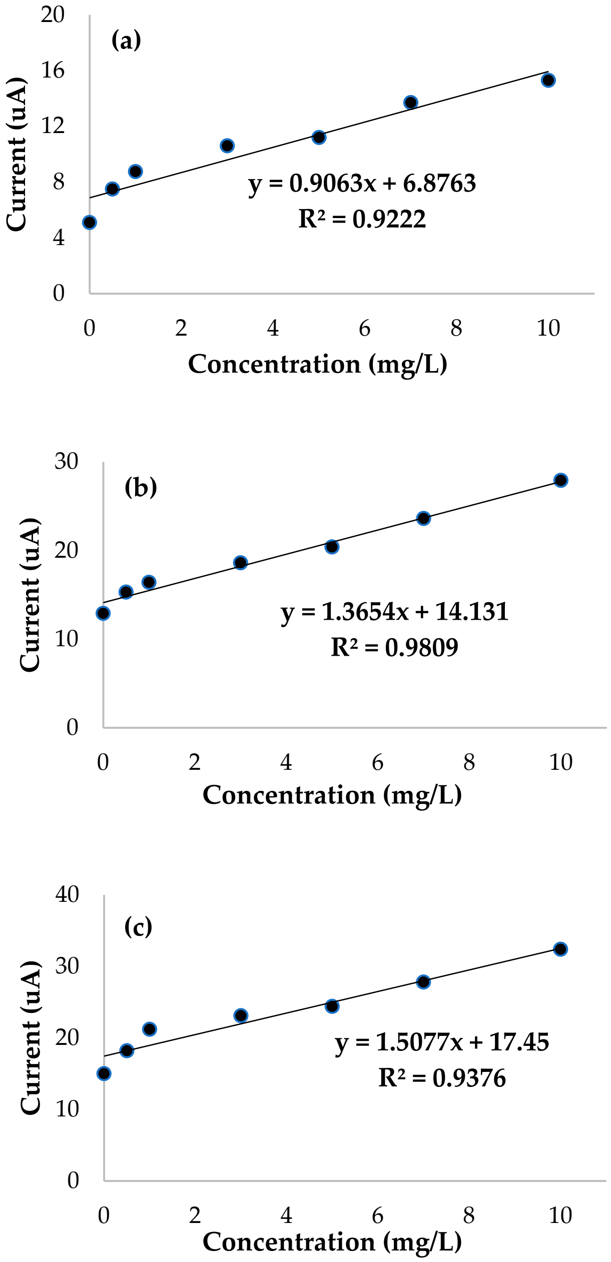

Figure S2. Calibration curves for the alginate biopolymer chemosensor measured by cyclic voltammetry: (**a**) 0.5%, (**b**) 1%, (**c**) 1.5%;

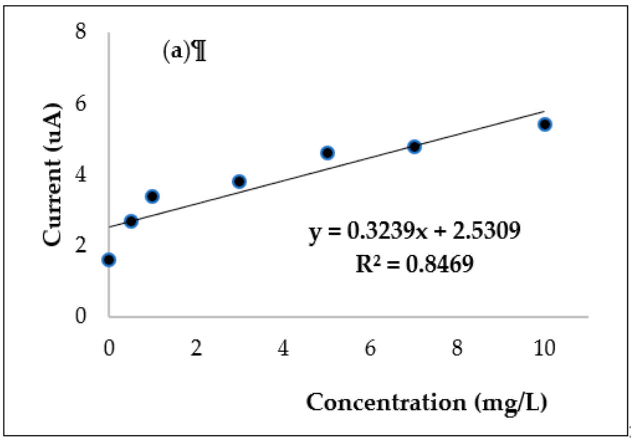

(a)

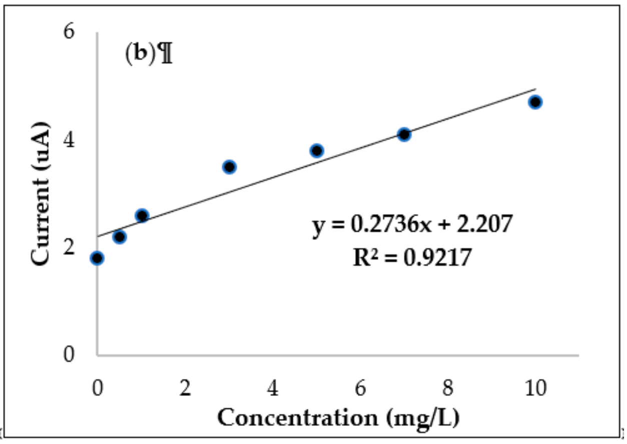

(b)

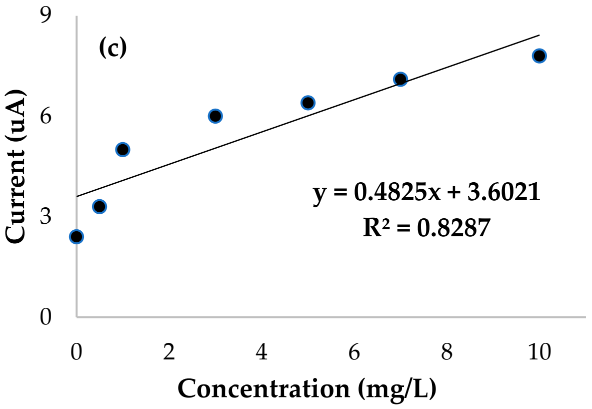

Figure S3. Calibration curves for the carrageenan biopolymer chemosensor measured by cyclic voltammetry: (**a**) 0.5%, (**b**) 1%, (c) 1.5%;

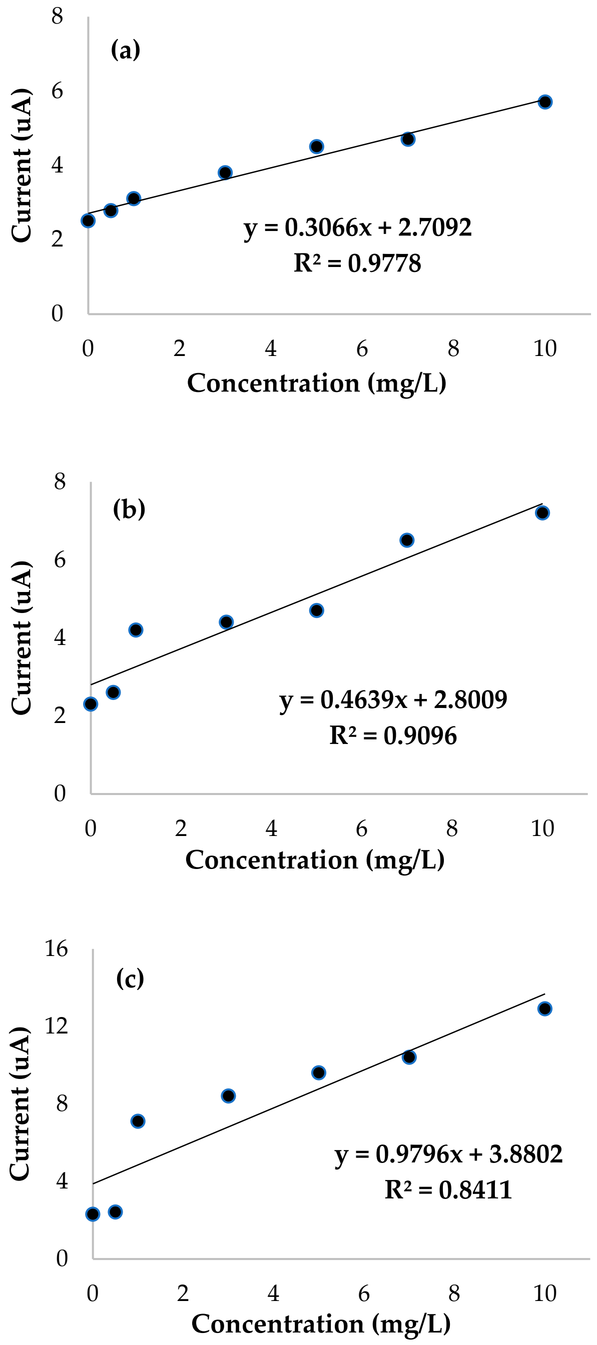

Figure S4. Calibration curves for the chitosan biopolymer chemosensor measured by cyclic voltammetry: (**a**) 0.5%, (**b**) 1%, (**c**) 1.5%;
